# Functional role of intrahepatic monocyte subsets for the progression of liver inflammation and liver fibrosis in vivo

**DOI:** 10.1186/1755-1536-5-S1-S27

**Published:** 2012-06-06

**Authors:** Frank Tacke

**Affiliations:** 1 Dept of Medicine III, University Hospital Aachen, Germany

## Abstract

Sustained inflammation upon chronic liver injury induces the development of liver fibrosis in mice and men. Experimental models of liver fibrosis highlight the importance of hepatic macrophages, so-called Kupffer cells, for perpetuating inflammation by releasing proinflammatory cytokines and chemokines as well as activating hepatic stellate cells (HSC). Recent studies in mice demonstrate that these actions are only partially conducted by liver-resident macrophages, classically termed Kupffer cells, but largely depend on recruitment of monocytes into the liver. Monocytes are circulating precursors of tissue macrophages and dendritic cells (DC), which comprise two major subsets in blood, characterized by the differential expression of chemokine receptors, adhesion molecules and distinct markers, such as Ly6C/Gr1 in mice or CD14 and CD16 in humans. Upon organ injury, chemokine receptor CCR2 and its ligand MCP-1 (CCL2) as well as CCR8 and CCL1 promote monocyte subset accumulation in the liver, namely of the inflammatory Ly6C^+ ^(Gr1^+^) monocyte subset as precursors of tissue macrophages. The infiltration of proinflammatory monocytes into injured murine liver can be specifically blocked by novel anti-MCP-1 directed agents. In contrast, chemokine receptor CX3CR1 and its ligand fractalkine (CX3CL1) are important negative regulators of monocyte infiltration in hepatic inflammation by controlling their survival and differentiation into functionally diverse macrophage subsets. In patients with liver cirrhosis, 'non-classical' CD14^+^CD16^+ ^monocytes are found activated in blood as well as liver and promote pro-inflammatory along with pro-fibrogenic actions by the release of distinct cytokines and direct interactions with HSC, indicating that the findings from murine models can be translated into pathogenesis of human liver fibrosis. Moreover, experimental animal models indicate that monocytes/macrophages and DCs are not only critical for fibrosis progression, but also for fibrosis regression, because macrophages can also degrade extracellular matrix proteins and exert anti-inflammatory actions. The recently identified cellular and molecular pathways for monocyte subset recruitment, macrophage differentiation and interactions with other hepatic cell types in injured liver may therefore represent interesting novel targets for future therapeutic approaches in liver fibrosis.

## Introduction

Chronic liver inflammation is a major health problem worldwide, eventually resulting in liver fibrosis, end-stage liver cirrhosis, organ failure, hepatocellular carcinoma and ultimately death. In industrialized countries, chronic hepatitis C, alcohol abuse and non-alcoholic steatohepatitis (NASH) are major causes for liver cirrhosis [1]. Liver fibrosis commonly develops as a result of chronic organ inflammation which is characterized by the infiltration and activation of immune cells, leading to both inflammatory as well as wound healing responses including subsequent necrosis and apoptosis of parenchymal cells and replacement by connective tissue and extracellular matrix (ECM) proteins. Although the different etiologies of liver fibrosis (e.g., alcohol abuse, chronic viral hepatitis, metabolic syndrome and autoimmune disorders) employ different mechanisms of hepatocyte injury, the resulting steps promoting fibrosis development have been found to be astonishingly similar despite the very different disease onsets [2].

Over recent years, several studies have emphasized the crucial role of infiltrating monocytes for the progression of liver inflammation and fibrosis in experimental mouse models [3-7]. It has become clear that the macrophage compartment of the liver, traditionally called 'Kupffer cells', is constantly replenished to a significant extent by blood monocytes [1,8] and is greatly augmented by an overwhelming number of infiltrating monocytes upon acute or chronic liver injury [4,9]. During fibrosis progression in mice, monocyte-derived macrophages can release several cytokines perpetuating chronic inflammation as well as directly activate hepatic stellate cells (HSC), resulting in their proliferation and transdifferentiation into collagen-producing myofibroblasts [3,4,7]. Nevertheless, it has to be kept in mind that liver-resident macrophages (Kupffer cells) may be capable of proliferating under certain conditions [10]. However, up to now this Kupffer cell proliferation has only be observed in inflammatory conditions that are extremely skewed towards "type 2" responses (T-helper cell 2/interleukin-4 driven conditions), such as parasitic infections [10], raising the question whether local macrophage proliferation could be of general relevance in liver fibrosis as well [11]. This review will discuss recent evidence from our laboratory about the functional role of monocyte subsets for liver inflammation and fibrosis as well as distinct chemokine actions driving monocyte migration and differentiation that may possibly represent novel therapeutic targets in hepatic fibrosis.

## Monocyte subsets in men and mice

Monocytes represent about 5-10% of peripheral blood leukocytes in humans and mice. They originate from a myeloid precursor in the bone marrow, circulate in the blood, bone marrow and spleen, and then enter tissues [12,13]. Monocytes are regarded as circulating precursors for tissue macrophages and dendritic cells (DCs). Migration of monocytes into tissues and differentiation into macrophages or DCs is believed to be largely determined by the inflammatory milieu, i.e. adhesion molecules, chemokines and pathogen-associated pattern-recognition receptors [12].

Heterogeneity among human monocytes was recognized over twenty years ago [14]. The differential expression of CD14 (part of the receptor for lipopolysaccharide) and CD16 (also known as FcγRIII) were used to define two major subsets in peripheral blood: 'classical' CD14^++^CD16^- ^monocytes, typically representing up to 95% of the monocytes in healthy individuals, and the 'non-classical' CD14^+^CD16^+ ^cells comprising the remaining fraction of monocytes [15]. These subsets differ in many respects, including adhesion molecule and chemokine receptor (CCR) expression. CD14^++^CD16^- ^monocytes express CCR2, CD62L (L-Selectin) and FcγRI (CD64), whereas CD14^+^CD16^+ ^monocytes lack CCR2, and have higher levels of MHC-II and FCγRII (CD32). Both subsets express the receptor for fractalkine, CX_3_CR1, but CD14^+^CD16^+ ^monocytes characteristically express higher levels [13,16].

More recently, it was noted that different monocyte subpopulations also exist in mice [17]. Based on similar adhesion molecule and chemokine receptor as well as similar gene expression profiles, murine Ly6C^hi ^(Gr1^hi^) monocytes are considered counterparts of human CD14^++^CD16^- ^monocytes, and murine Ly6C^lo ^(Gr1^lo^) may represent the subpopulation comparable to human CD16^+ ^monocytes [16]. More recently, human CD16^+ ^monocytes have been further subdivided into 'intermediate' CD14^+^CD16^+ ^and 'non-classical' CD14^dim^CD16^++ ^cells [18], with the latter likely sharing important functional similarities to murine Ly6C^lo ^monocytes [19].

## Migratory and functional differences of monocyte subsets

Based on a distinct pattern of chemokine receptors expressed on their surface, both monocyte subsets show fundamentally different patterns in their migratory behavior. Namely, the chemokine receptors CCR1 and CCR2 are more highly expressed on Ly6C^hi ^mouse monocytes, whereas CCR5 and CX_3_CR1 are elevated on murine Ly6C^lo ^monocytes [13,16,20]. Both subsets constitutively patrol the blood vessels of the entire circulation and also to some extent the secondary lymphatic system. Monocytes therefore are able to act as 'first responders' to inflammatory signals, infiltrating inflamed tissues shortly after the onset of injury. This process has mainly been observed for Ly6C^hi ^monocytes and is highly dependent on chemokine receptors CCR2 and CCR6 [21-23]. CCR2 has been described to mediate release of Ly6C^hi ^monocytes from the bone marrow as well as driving tissue infiltration directly [20,24-26], whereas CCR6 (ligand: CCL20) seems to be of some importance for guiding infiltrating cells to the site of injury once the monocytes have left the bloodstream e.g. in inflamed skin areas [23,27,28]. In injured liver, the chemokine receptor CCR8 appears to be also critical for Ly6C^hi ^monocyte accumulation and subsequent pro-inflammatory macrophage differentiation *in vivo *[29]. Ly6C^hi ^monocytes are rapidly recruited to sites of inflammation, such as in atherosclerosis, peritonitis, urinary tract infection or after organ damage into the injured liver [4,20,24,30,31]. Within the inflamed tissue, Ly6C^hi ^monocytes were found to give rise to pro-inflammatory macrophages and TNF-producing DCs in inflamed tissue [4,12,20,26,31].

In contrast, Ly6C^lo ^monocytes appear to have a more patrolling behavior at the endothelium [32]. Imaging studies using intravital microscopy in dermis tissue suggested that Ly6C^lo ^cells scarcely leave blood vessels without inflammatory stimuli, but rather showed a patrolling behaviour rolling along endothelia in a LFA-1 and CX_3_CR1 dependent fashion [17]. Ly6C^lo ^monocytes have also been found to migrate into the inflamed intima in atherosclerotic plaques where they preferentially differentiate into 'non-classical' CD11c^+ ^macrophages [20]. Other studies indicated that Ly6C^lo ^monocytes may serve as precursors for alternatively activated macrophages, possibly fulfilling functions in tissue repair and resident macrophage/DC turnover [12,32,33]. A natural reservoir for Ly6C^lo ^monocytes *in vivo *appears to be the spleen [34].

The origin of 'non-classical' Ly6C^lo ^monocytes is controversially debated among monocyte researchers. It is apparent that Ly6C^lo ^monocytes can develop from Ly6C^hi ^monocytes as a more mature subset [35,36], and the 'conversion' from Ly6C^hi ^to Ly6C^lo ^monocytes likely takes place in the bone marrow under homeostatic conditions [36]. However, experimental data exist indicating that Ly6C^lo ^monocytes may also in parallel arise from distinct precursors in the bone marrow, independent from the Ly6C^hi ^subset [12,30,37]. From experimental hepatological research, very limited data exist about the functional role of Ly6C^lo ^monocytes, but it has to be mentioned that infiltrating, inflammatory Ly6C^hi ^monocytes rapidly downregulate Ly6C surface expression upon transmigration into (injured) liver [4,38].

## Role of CCR2 and CCR8 for monocyte infiltration in liver fibrosis

Accumulating evidence from murine models demonstrated that the infiltration of monocytes into the liver is a major pathogenic factor for chronic hepatic inflammation and fibrosis [3-6]. In order to identify the role of monocyte subsets in hepatic fibrosis, we characterized subpopulations of infiltrating monocytes in acute and chronic carbon tetrachloride (CCl_4_)-induced liver injury in mice using flow-cytometry and immunohistochemistry [4]. Inflammatory Ly6C^hi^, but not Ly6C^lo ^monocytes were massively recruited into the liver upon toxic injury constituting an up to 10-fold increase in CD11b^+^F4/80^+ ^intrahepatic macrophages early (24-48 hours) after CCl_4 _injection and persistently a 3- to 5-fold increase in fibrotic livers at 6 weeks after biweekly CCl_4 _injections. During chronic liver damage, intrahepatic CD11b^+^F4/80^+^Ly6C^+ ^monocyte-derived cells differentiated preferentially into iNOS-producing macrophages exerting proinflammatory and profibrogenic actions, e.g. promoting hepatic stellate cell (HSC) activation, T_H_1-T cell differentiation and transforming growth factor beta (TGFβ)-release [4].

**Figure 1:**
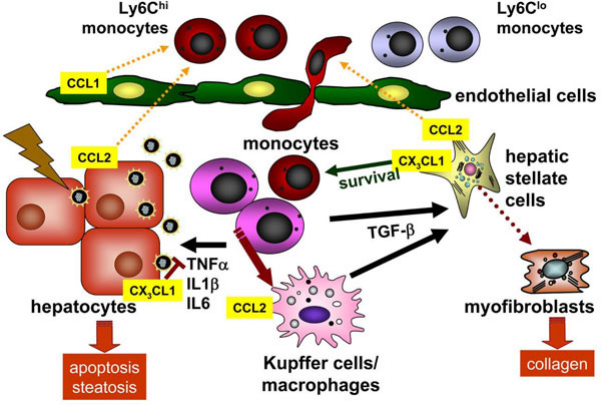
**Monocyte subsets in experimental liver fibrosis in mice**. Upon liver injury, hepatocytes, hepatic stellate cells, endothelium and resident macrophages (Kupffer cells) release many cytokines and chemokines (not shown in detail). CCL2 is an important mediator that promotes the accumulation of inflammatory CCR2^+ ^Ly6C^hi ^(Gr1^hi^) monocytes from blood into the injured liver. CCR8 and its ligand CCL1 also promote monocyte/macrophage migration in hepatic fibrosis. Intrahepatic monocytes develop preferentially into 'M1' inflammatory macrophages in this setting. These monocytes/macrophages release proinflammatory cytokines such as TNFα, IL1β or IL6 that further promote hepatocyte apoptosis, hepatocyte steatosis and inflammation, but also directly interact with hepatic stellate cells via TGFβ, thereby contributing to myofibroblast transdifferentiation and collagen production. CX_3_CL1-CX_3_CR1 interactions on monocytes/macrophages are anti-inflammatory and anti-fibrotic by prolonging monocyte survival and limiting M1 macrophage differentiation.

Because Ly6C^hi ^monocytes express high levels of CCR2 [20] and activated hepatic stellate cells were shown to release high amounts of the CCR2-ligand MCP-1 [39,40], we compared wildtype with CCR2- and CCR2/CCR6-deficient mice in the CCl_4 _fibrosis model in order to identify possible chemokine receptor pathways mediating monocyte migration into the liver. These experiments revealed that the chemokine receptor CCR2 critically controls intrahepatic Ly6C^hi ^monocyte accumulation, at the level of mediating their egress from the bone marrow. Impaired monocyte subset recruitment in CCR2^-/- ^and CCR2^-/-^CCR6^-/- ^mice resulted in reduced HSC activation and diminished liver fibrosis. Moreover, adoptively transferred Ly6C^hi ^monocytes traffic into the injured liver and promote fibrosis progression in wildtype and CCR2^-/-^CCR6^-/- ^mice, which are otherwise protected from hepatic fibrosis. Intrahepatic CD11b^+^F4/80^+^Ly6C^+ ^monocyte-derived macrophages purified from CCl_4_-treated animals, but not 'naïve' bone marrow monocytes or control lymphocytes, directly activate HSC in a TGFβ-dependent manner *in vitro *[4]. Collectively, these data indicate that inflammatory Ly6C^+ ^monocytes, recruited into the injured liver via CCR2-dependent bone marrow egress, promote the progression of liver fibrosis (Figure 1).

Several independent studies highlighted the importance of the chemokine receptor CCR2 and its cognate ligand monocyte-chemoattractant protein 1 (MCP-1/CCL2) for monocyte recruitment during experimental hepatic fibrosis [3-6], suggesting that inhibition of CCR2 or MCP-1 might bear therapeutic potential in hepatic fibrosis. Therefore, we have investigated the pharmacological inhibition of MCP-1 via the structured L-enantiomeric RNA oligonucleotide mNOX-E36 (a so-called Spiegelmer) that potently binds and inhibits murine MCP-1 in two murine models of chronic liver diseases [41]. Antagonizing MCP-1 by mNOX-E36 efficiently inhibited murine monocyte chemotaxis *in vitro *as well as migration of Ly6C^+ ^blood monocytes into liver upon injury *in vivo*. In line with lower levels of intrahepatic macrophages, pro-inflammatory cytokines (TNF, IFNγ, IL-6) were significantly reduced in injured liver tissue, and the development of steatosis and steatohepatitis was ameliorated by anti-MCP-1 treatment [41].

Moreover, CCR1 and CCR5, receptors for the chemokines CCL3/MIP1α, CCL4/MIP1β and CCL5/RANTES, were found to promote liver fibrosis in mice [9]. Of note, while the function of CCR1 could be attributed to infiltrating immune cells (most likely Ly6C^+ ^monocytes), CCR5 appears to exert its actions primarily on resident hepatic non-parenchymal cells, most probable on HSC [9]. Recent experimental data from our laboratory indicate that the chemokine receptor CCR8 (main ligand: CCL1) is also involved in directing infiltration of inflammatory monocytes into injured liver and in promoting preferential differentiation into macrophages with a pro-inflammatory phenotype [29]. CCR8-deficient mice showed significantly reduced hepatic fibrosis in two independent experimental models, which was accompanied by reduced intrahepatic monocytes/macrophages and could be restored by adoptively transferring CCR8-expressing Ly6C^hi ^bone marrow monocytes [29].

## Role of CX_3_CR1 for macrophage differentiation in liver fibrosis

The chemokine receptor CX_3_CR1 is expressed at high levels on both monocyte subsets (Ly6C^lo^>>Ly6C^hi^) and has been identified as an important regulator of monocyte migration, differentiation and survival [13]. Preliminary observations have linked CX_3_CR1 and its ligand fractalkine (CX_3_CL1) to the pathogenesis of chronic liver diseases. As such, fractalkine and CX_3_CR1 were found up-regulated in biopsies of patients with acute and chronic liver injury [42], especially in cholestatic diseases [43,44]. Furthermore, CX_3_CR1 gene polymorphisms have been associated with fibrosis progression in patients with chronic hepatitis C [45]. Experimentally, shedding of CX_3_CL1 by HSC promoted chemoattraction of monocytes *in vitro *[46], and the adhesion of human CD16^+ ^monocytes to liver sinusoidal endothelium was partially mediated by CX_3_CR1 [47]. We therefore set out experiments to define the role of fractalkine and CX_3_CR1 for liver inflammation and fibrosis.

To analyze the functional relevance of this pathway, two models of experimental liver fibrosis were applied to wildtype (WT) and CX_3_CR1-deficient mice, namely repetitive carbon tetrachloride (CCl_4_) injections as well as surgical ligation of the biliary duct (BDL, bile duct ligation) [48]. Upon liver injury in mice, fractalkine expression was induced, primarily in hepatocytes and hepatic stellate cells (HSC). CX_3_CR1^-/- ^developed greater hepatic fibrosis than WT animals in CCl_4_- and BDL-induced fibrosis. Surprisingly, CX_3_CR1^-/- ^mice displayed significantly increased numbers of monocyte-derived macrophages within the injured liver. Bone marrow transplanted chimeric animals revealed that CX_3_CR1 restricts hepatic fibrosis progression and monocyte accumulation through mechanisms exerted by infiltrating immune cells. In the absence of CX_3_CR1, intrahepatic monocytes developed preferentially into pro-inflammatory TNF- and iNOS-producing macrophages (Figure 1) [48]. This impact on differentiation has been also observed by *in-vitro *experiments, which demonstrated increased TNF and reduced arginase-1 expression by CX_3_CR1-deficient hepatic macrophages upon CCl_4 _stimulation [49].

Although CX_3_CL1 was originally defined as a chemoattractant for monocytes [20,50] a growing body of evidence indicated that CX_3_CR1 is involved in controlling cell survival. In conditions of hepatic inflammation and fibrosis, CX_3_CR1 represents an essential survival signal for hepatic monocyte-derived macrophages by activating anti-apoptotic *bcl-2 *expression [48]. Monocytes/macrophages lacking CX_3_CR1 undergo increased cell death following liver injury, which then perpetuates inflammation, promotes prolonged inflammatory monocyte infiltration into the liver and results in enhanced liver fibrosis [48]. Taken together, CX_3_CR1 limits liver fibrosis *in vivo *by controlling differentiation and survival of intrahepatic monocytes (Figure 1). Pharmacological augmentation of this pathway may thus represent a possible therapeutic antifibrotic strategy [51].

## Role of monocyte subsets for fibrosis regression

Although infiltrating monocytes/macrophages have clearly been shown to contribute to the progression of fibrosis *in vivo *(see above), they appear to be capable of acting as important beneficial contributors to fibrosis regression as well [51]. The selective depletion of macrophages, using a transgenic mouse model with diphtheria toxin receptor expression on myeloid (CD11b^+^) cells, during the resolution phase after CCl_4_-induced injury significantly impaired resolution of fibrosis [7]. This effect could be mainly attributed to reduced expression of matrix metalloproteinase (MMP) 13 (collagenase 3) by scar-associated macrophages, which is the key enzyme to degrade excessive extracellular matrix proteins [52]. Moreover, macrophage derived elastase MMP-12 regulates the degradation of elastin, which is a major ECM component characterizing advanced fibrosis/cirrhosis [53]. It is very likely that these beneficial actions are largely conducted by specifically differentiated monocyte-derived cells, because the adoptive transfer of macrophage subsets into (regressing) experimental fibrosis was shown to improve hepatic fibrosis in mice [54]. Similarly, distinct subpopulations of myeloid (likely monocyte-derived) dendritic cells have been observed during fibrosis regression, which also functionally facilitated hepatic fibrosis regression upon adoptive transfer experiments in mice [55]. However, the exact phenotype of infiltrating monocytes and monocyte-derived cells during fibrosis regression and its potential for cell-based therapeutic approaches in humans demands further investigations.

## Translation of findings from murine models into human pathogenesis

The recent advances in understanding the role of monocyte subsets and macrophage actions in murine experimental hepatic fibrosis raised the question whether these mechanisms can be directly translated into human pathogenesis. CCL2-CCR2 interactions had long been suggested as an important mediator of macrophage accumulation in human liver diseases as well [56]. As the interference with monocyte subset infiltration, differentiation and activation may represent an interesting novel target for future therapeutic approaches in liver fibrosis [1], our laboratory aimed at defining the functional contributions of monocyte subpopulations to liver fibrogenesis in human liver disease as well. We therefore analyzed circulating monocyte subsets from freshly drawn blood samples of 226 patients with chronic liver disease (CLD) and 184 healthy controls by FACS analysis [57]. Circulating monocytes were significantly expanded in CLD-patients compared to controls with a marked increase of the 'non-classical' CD14^+^CD16^+ ^subset. These CD14^+^CD16^+ ^monocytes showed an activated phenotype in patients (e.g., increased HLA-DR expression) and correlated with proinflammatory cytokines and clinical progression. Correspondingly, CD14^+^CD16^+ ^macrophages massively accumulated in fibrotic/cirrhotic livers, as evidenced by immune-fluorescence and FACS from biopsies and explanted cirrhotic livers. Ligands of monocyte-related chemokine receptors CCR2, CCR1 and CCR5 were expressed at higher levels in fibrotic and cirrhotic livers, while CCL3 and CCL4 were also systemically elevated in CLD-patients [57]. Fractalkine was significantly up-regulated in the circulation upon disease progression, while CX_3_CR1 was down-regulated intrahepatically in patients with advanced liver fibrosis or cirrhosis [48]. However, it has to be kept in mind that despite many similarities the chemokine system between mice and men do not fully overlap. As such, interleukin-8 (IL-8, CXCL8), a potent chemoattractant for neutrophils and ligand for the receptors CXCR1 and CXCR2, has no direct homologue in mice. IL-8 is strongly upregulated in the livers and the serum of patients with liver fibrosis [58]. Interestingly, IL-8 does not seem to primarily promote neutrophil attraction during fibrogenesis, but rather contributes to accumulation of monocytes in fibrotic livers, as circulating monocytes in patients with cirrhosis significantly upregulate CXCR1 on their surface [58].

We also isolated human monocyte/macrophage subpopulations from healthy volunteers and patients with liver cirrhosis to functionally characterize these subsets regarding cytokine/chemokine expression and interactions with primary human hepatic stellate cells (HSC) *in vitro*. CD14^+^CD16^+ ^monocytes released abundant proinflammatory cytokines. Furthermore, CD14^+^CD16^+^, but not CD14^+^CD16^- ^monocytes could directly activate collagen-producing HSC [57].

Given the assumption that CD14^+^CD16^+ ^monocytes would resemble Ly6C^lo ^cells in mice [13], our findings reveal a considerable discrepancy in human disease from mouse models, because fibrosis induction and progression in mice is accompanied by Ly6C^hi ^monocytosis in peripheral blood and infiltration of Ly6C^hi ^monocytes into the injured liver [4]. The reasons for this discrepancy are currently unknown. One obvious difference between murine models and the human diseased liver is the strikingly dissimilar time-course of fibrosis development. Whereas experimental murine fibrosis is analyzed at three or six weeks after induction, e.g. by BDL or CCl_4 _injection, human fibrosis and cirrhosis usually develops over decades of chronic injury and inflammation, and the most prominent enrichment of CD14^+^CD16^+ ^monocytes were apparent in end-stage cirrhosis [57]. On the other hand, the assumption that CD14^+^CD16^+ ^human monocytes are equivalents of murine Ly6C^lo ^monocytes is primarily based on conserved gene and protein profiles between these subsets [16], but not on functional assays. In more functional approaches, only CD14-low expressing CD16^+ ^monocytes (so called CD14^dim ^cells) displayed characteristics of murine Ly6C^lo ^monocytes [19]. Our experiments demonstrated that murine Ly6C^hi ^monocyte-derived cells in inflammatory conditions and human CD14^+^CD16^+^-derived macrophages share important functional properties, particularly the expression of pro-inflammatory cytokines such as TNFα or nitric oxide, and the ability to directly activate HSC [4,57].

With respect to human hepatofibrogenesis, our data demonstrate the expansion of CD14^+^CD16^+ ^monocytes in the circulation and liver of CLD-patients upon disease progression and suggest their functional contribution to the perpetuation of intrahepatic inflammation and profibrogenic HSC activation in liver cirrhosis. The modulation of monocyte-subset recruitment into the liver via chemokines/chemokine receptors and their subsequent differentiation may therefore indeed represent promising approaches for therapeutic interventions in human liver fibrosis [51].

## Conclusions

Monocytes are circulating cellular precursors for macrophages and dendritic cells. In mice and men, monocyte subpopulations have been identified that differ with respect to migratory and functional behaviour. Accumulating experimental evidence recently emphasized that the infiltration of inflammatory monocyte subsets is a key factor for the progression of hepatic inflammation and fibrosis in injured murine liver. CCR2 and CX_3_CR1 have been identified as critical, partially counteracting chemokine pathways regulating monocyte migration and differentiation in hepatofibrogenesis. In patients with chronic liver diseases and cirrhosis, non-classical CD14^+^CD16^+ ^monocytes exert pro-inflammatory and pro-fibrogenic actions. The therapeutic targeting of monocyte/macrophages by interfering with their migration (chemokine inhibitors) and differentiation or by direct cell transplantation during progression and regression of fibrosis are currently under intense investigation.

## List of abbreviations

BDL: bile duct ligation; BM: bone marrow; CCR: C-C motif chemokine receptor; CCL: C-C- motif chemokine; CCl_4_: carbon tetrachloride; CX_3_CL1: fractalkine; CX_3_CR1: fractalkine receptor; FACS: fluorescence activated cell sorting; Gr1: myeloid cell marker; HBV: hepatitis B virus; HCV: hepatitis C virus; HSC: hepatic stellate cell; IL: interleukin; Ly6C: myeloid cell marker; MCP-1: monocyte chemoattractant protein-1; SMA: smooth muscle actin; TGFβ: transforming growth factor beta; TNF: tumor necrosis factor.

## Competing interests

The laboratory of Frank Tacke has received funding in the past from Noxxon Pharma GmbH, Berlin, Germany.
